# Room-Temperature, Ionic-Liquid-Enhanced, Beta-Cyclodextrin-Based, Molecularly Imprinted Polymers for the Selective Extraction of Abamectin

**DOI:** 10.3390/nano12061017

**Published:** 2022-03-20

**Authors:** Saqib Farooq, Bochang Chen, Shakeel Ahmad, Ihsan Muhammad, Quaid Hussain, Haiyan Wu

**Affiliations:** 1 Guangxi Key Laboratory of Agric-Environment and Agric-Products Safety, Agricultural College of Guangxi University, Nanning 530004, China; saqibhort@gmail.com (S.F.); 15677802394@163.com (B.C.); shakeel1287@hotmail.com (S.A.); ihsanagrarian@yahoo.com (I.M.); 2 State Key Laboratory of Subtropical Silviculture, Zhejiang A&F University, 666 Wusu Street, Hangzhou 311300, China; quaid_hussain@yahoo.com

**Keywords:** abamectin, room-temperature ionic liquid, molecularly imprinted polymers, selective extraction, pesticide residues

## Abstract

To ensure environmental protection and food quality and safety, the trace level detection of pesticide residues with molecularly imprinted polymers using a more economic, reliable, and greener approach is always demanded. Herein, novel, enhanced, imprinted polymers based on beta-cyclodextrin, using room-temperature, ionic liquid as a solvent for abamectin were developed with a simple polymerization process. The successful synthesis of the polymers was verified, with morphological and structural characterization performed via scanning electron microscope analysis, nitrogen adsorption experiments, and thermogravimetric analysis. The imprinted polymers showed good adsorption ability, which was confirmed with a pseudo-second-order kinetic model and a Langmuir isotherm model, as they exhibit a theoretical adsorption of 15.08 mg g^−1^ for abamectin. The polymers showed high selectivity for abamectin and significant reusability without significant performance loss. The MIPs were used to analyze abamectin in spiked apple, banana, orange, and grape samples, and as a result, a good recovery of 81.67−101.47%, with 1.26−4.36% relative standard deviation, and limits of detection and quantitation of 0.02 µg g^−1^ and 0.05 µg g^−1^, respectively, was achieved within a linear range of 0.03−1.50 µg g^−1^. Thus, room-temperature, ionic-liquid-enhanced, beta-cyclodextrin-based, molecularly imprinted polymers for the selective detection of abamectin proved to be a convenient and practical platform.

## 1. Introduction

Economic agricultural production is widely dependent on pesticide application to ensure livelihood; however, the application of such agrochemicals has various consequences and detrimental effects on the food chain [1]. To minimize such adverse effects, optimized application methods of pesticides have regularly been developed [2]; still, pesticide residues at trace level should be constantly monitored because their detection at trace levels is a very complex and tiresome job, as it requires a lot of pre-treatment extraction techniques, including reducing matrix interference, and high precision, especially in fruit and vegetable samples.

Over the past decade, molecularly imprinted polymers (MIPs) have received multifield applications [3,4] and have also played a vital role in food quality and safety [5,6]. The synthesized, 3D-imprinted materials provide fascinating and qualitative analytical methods [7,8] with wide adaptability [9], low cost [10], high sensitivity, robustness, and selectivity [11,12]. Still, the application of MIPs in actual sample analysis is very limited owing to certain hindrances, like eco-safety, low commercial application, limited applicability, and complex preparation with lower efficiency [13,14,15]. To ameliorate such shortcomings, the selective extraction of an analyte with efficient MIPs is necessary. The application of MIPs and MIP-based sensors is an advanced analytical approach for pesticide residue detection [16,17]. Beta-cyclodextrin (β-CD) has a fascinating ability for making firm, non-inclusion complexes with analytes due to its unique molecular structure having a hydrophilic exterior and a hydrophobic interior [18]. Cyclodextrin, as an acting, functional monomer in MIP synthesis. easily creates a reversible host–guest interaction and improvises to develop efficient MIPs due to its firm adaptability and eco-friendly nature, as compared to traditional MIPs [19,20]. Room-temperature, ionic liquids (RTILs) have prime abilities, including a highly tunable nature [21], wide applicability [22], thermostability [23], and ionic conductivity [24], which endow them as “designer solvents” [25]. The literature provides evidence that MIPs synthesized using RTILs as solvents are more efficient, as they provide a compatible medium for ionic interaction [26,27]. 1,6-hexamethylene diisocyanate (HMDI) is a well-known cross-linker which belongs to an aliphatic diisocyanate group that interacts with guest molecules via hydrogen bonding [28].

**Figure 1:**
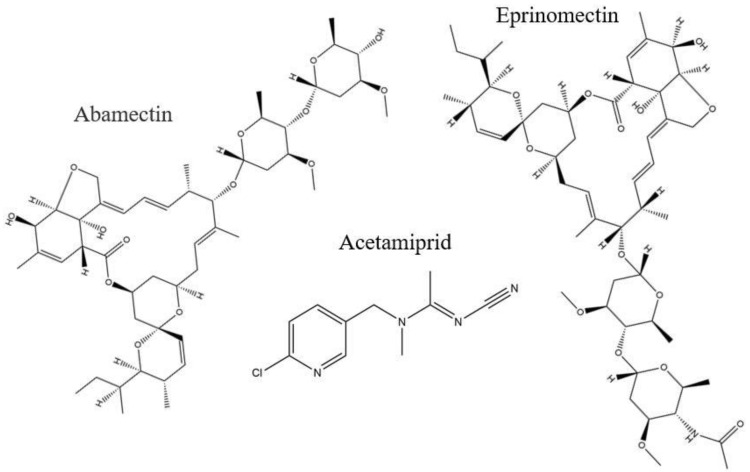
Chemical structures of abamectin, acetamiprid, and eprinomectin.

Among avermectins, abamectin (ABM) (Figure 1) is considered highly toxic [29] and is produced both naturally and synthetically [30]. It is used as a nematicide, acaricide, and pesticide in fruits, vegetables, and other crops; therefore, with such a wide range of applications, it poses a potential risk to the ecological environment. ABM causes hyperexcitability and neurotransmission in insects as it blocks chloride channels and is a GABA-receptor inhibitor [31]. ABM has acute toxicity to both non-target and target organisms [32], causing neurological impairment in animals, minimizing immunity to enzymatic activities, and being genotoxic and hepatotoxic in human hepatocyte cells [33], and it is also pest resistant [34,35]. Previously, different methods were developed for ABM-detection, such as high-performance liquid chromatography with ultraviolet detection (HPLC–UV) [36], enzyme-linked immunosorbent assay (ELISA) [37] liquid chromatography–tandem mass spectrometry (LC–MS) [38], and high-performance liquid chromatography–fluorescent detection (HPLC–FLD) [39], but such methods lack precision or selectivity, or they exhibit laborious, pre-treatment techniques, and they have limited application to specific samples. The objective of this work was to develop a selective extraction method for ABM with β-CD-based MIPs, while enhancing performance by using an ionic liquid which has not been previously reported for use with abamectin. Before the analysis of real fruit samples with the developed MIPs using HPLC-UV, all the analytical parameters were optimized.

## 2. Materials and Methods

### 2.1. Chemicals and Reagents

ABM (97%), acetamiprid (99%), eprinomectin (91%), HPLC-grade methanol, acetonitrile, acetic acid, and acetone were obtained from Macklin Biochemical Technology Co., Ltd. (Shanghai, China). HMDI (>98%) and 1-butyl-3-methylimidazolium tetrafluoroborate ([BMIM]BF_4_) (>98%) were acquired from TCI (Shanghai, China). β-CD (>98%) was provided by Sinopharm Chemical Reagent Co. Ltd. (Beijing, China), and before utilization, it was dried for 24 h at 110 °C. Dimethyl sulfoxide (DMSO) (Superdry) (≥99.9%) was provided by J&K Tech. Ltd. (Beijing, China). Fruits samples were purchased from the local market in Nanning, Guangxi (China). All the reagents used were of analytical grade.

### 2.2. Instrumentation and Chromatographic Analysis

Ultrapure water was obtained from Milli-Q IQ 7000 (Merck, Darmstadt, Germany). The scanning electron microscope (SEM) Zeiss GeminiSEM 500 (Oberkochen, Germany), Tristar II 3020 (Micromeritics, Norcross, USA), and TGA/DSC 1/1600 (Mettler Toledo, Schwerzenbach, Switzerland) were used for structural analysis. The other instruments used during this experiment were a magnetic stirrer DF-101D (Yuhua, Changzhou, China); a thermostatic oscillator HZQ-F160A (Shanghai Yiheng scientific instruments, Shanghai, China); a Heraeus Vacutherm VT 6060M (Thermo Electron, Langenselbold, Germany); a Mix-30S vortex mixer (Hangzhou MIU Instruments, China); a BY-400C centrifuge (Baiyang Medical Inst., Beijing, China): a 0.22 µm, 13 mm Nylon filter (Bojin, Tianjin, China); and a GT SONIC-D20 ultrasonic cleaner (Shenzhen, China).

The chromatographic analysis was performed on an HPLC Agilent^®^ Model 1260 Infinity II (Agilent Technologies, Santa Clara, CA, USA) with a binary pump (up to 600 bar) and an ultraviolet (UV) detector. The ZORBAX Eclipse Plus column (C18, 95Å, 4.6 × 150 mm, 5 µm) and Agilent OpenLAB Chromatography Data System software (Santa Clara, CA, USA) were used for the collection and analysis of the data. The mobile phase was composed of methanol/acetonitrile/water (27:55:18 *v*/*v*) which was degassed for 30 min before use. The flow rate was 1.2 mL min−1,250 nm wavelength, column temperature 25 °C, and injection volume of 20 µL [40]. The statistical analysis was performed with OriginPro, Version 2021 (OriginLab Corporation, Northampton, MA, USA).

### 2.3. Preparation of Molecularly Imprinted Polymers

The MIPs were prepared according to Long et al. [41]. First, ABM and β-CD were dissolved in DMSO/RTIL at 25 °C with N_2_ purging and stirred for two hours. HMDI was added before the temperature was raised to 65 °C and was continued for 8 h under N_2_ protection in a silicon oil bath. The prepared, unrefined MIPs were precipitated in acetone and were washed with methanol/acetic acid (9:1 *v*/*v*) and methanol and were dried at 50 °C. As a control, the non-imprinted polymers (NIPs) were made using the same method without template addition. The preparation protocols for ABM MIP synthesis are given in Table 1 and details of the polymerization components are given in Table S1.

### 2.4. Characterization Study

The structural analysis was performed with SEM operated at an accelerated voltage of 2 kV. Thermal stability was evaluated by thermogravimetric analysis (TGA) with a test temperature ranging from room temperature to 800 °C, with a heating rate of 10 °C/min, and nitrogen was used as the carrier gas. A gas adsorption experiment was carried out for the surface area using the Brunauer–Emmett–Teller (BET) test. The samples were degassed at 150 °C under vacuum for 12 h. The specific surface areas of the samples were calculated by BET; the pore volume (V_P_) and mean pore diameter (D_P_) were calculated by using the Barrett–Joyner–Halenda (BJH) model. The swelling analysis was carried out by packing 50 mg of polymers in graduated syringes of 1.0 mL and filling them with deionized water. The non-adsorbed water was removed after an equilibration time of 6 h at room temperature, and the mass of the swollen polymer was determined using the following Equation (1):

(1) Sr=(Me−Mi)/Mi

where *Sr* is the swelling ratio, *Me* is the mass of the swollen polymer, and *Mi* is the mass of the dry polymer.

### 2.5. Adsorption Measurements of MIPs

#### 2.5.1. Adsorption Kinetic Test

To determine the kinetic equilibrium time, 40 mg of dried MIPs or NIPs was mixed with 5 mL of standard concentration of ABM at 50 µg mL^−1^ in methanol for discrete time intervals ranging from 5 to 180 min. After being shaken at a specified time interval and incubated at 25 °C, the polymers were removed via centrifugation for 2 min at 8000 rpm. The supernatant was filtered with a 0.22 μm microporous membrane before HPLC–UV analysis to determine the unbound ABM.

The adsorption amount (*Q_t_*) (mg g^−^^1^) at a time (t) was derived by using the following equation [42]:

(2) $$Q_{t}=\frac{\left(C_{0}-C_{i}\right)\times V}{m}$$

where *C_0_* and *C_i_* are the initial and final ABM concentrations, respectively, solution volume is expressed at *V* (mL), and *m* expresses the weight of the polymers.

#### 2.5.2. Adsorption Isotherm Test

To evaluate the binding isotherms of the polymers towards ABM, they were studied by subjecting 40 mg of MIPs or NIPs to 5 mL of methanol solution of ABM at concentration levels ranging from 10 to 200 µg mL^−1^. The samples were shaken for equilibrium time at 25 °C and centrifuged at 8000 rpm for 2 min. For unbound ABM amounts, the supernatant was filtered and transferred to an HPLC–UV analysis. The ABM amount bound to polymers at equilibrium concentration (*Q_e_*, µg mL^−1^) was obtained by using the following Equation (3):

(3) $$Q_{e}=\frac{\left(C_{0}-C_{e}\right)\times V}{m}$$

where solution volume is expressed as *V* (mL), *C_0_* and *C_e_* are the initial and equilibrium concentrations of ABM (µg mL^−1^), and *m* expresses the weight of the polymers.

### 2.6. Selectivity Test

For investigation of the relative selective adsorption of the MIPs, two competitor compounds were selected, one as the structural analogue “Eprinomectin”, and one as the non-structural analogue “Acetamiprid”. For the MIPs and NIPs, 40 mg of each were added to 5 mL methanol standard solution (50 µg mL^−1^) of ABM, eprinomectin, and acetamiprid, separately. The samples were agitated for an equilibrium time of 30 min at 25 °C, centrifuged, filtered, and the supernatant was transferred to HPLC–UV for the unbound amount of each analyte analysis.

### 2.7. Template Extraction

The extraction of template molecules from the polymeric matrix is a crucial step, for which two different methods were studied for the purpose of comparison. 

Method 1: Soxhlet extraction was used to remove the template from the MIPs by using two types of eluents: (i) methanol/acetic acid and (ii) acetone/acetic acid with a ratio of 9:1 (*v*/*v*) (200 mL) each for about 12 h for each cycle; afterwards, the extract was concentrated with a rotatory evaporator and reconstituted in methanol, filtered, and subjected to HPLC–UV to analyze the extraction percentage until no template residues were found. The residual acetic acid was removed from the MIPs with 200 mL of pure methanol for 12 h and finally dried at 50 °C before storage at ambient temperature.

Method 2: The ultrasonication approach was used to clean the synthesized polymers by dispersing them in 10 mL methanol/acetic acid or acetone/acetic acid at a ratio of 9:1 (*v*/*v*) and sonicated for 3 min each time. Then, the supernatant was concentrated with a rotatory evaporator, and it was reconstituted in methanol, filtered, and subjected to HPLC–UV to analyze the extraction percentage until no template residues were found. Pure methanol was used to remove the residual acetic acid from the polymers, and they were dried at 50 °C before being stored at ambient temperature.

### 2.8. Reusability

To examine the applicability potential, binding and rebinding experiment cycles were carried out by suspending 40 mg of MIPs to 5 mL of ABM standard solution (50 µg mL^−1^) and agitating for 30 min to obtain maximum saturation. Then, the samples were centrifuged, and ABM was eluted according to method 2. The extract was concentrated and reconstituted in methanol, filtered, and subjected to HPLC–UV to analyze the bound amount of ABM for each cycle. 

### 2.9. Extraction of ABM from Real Samples

To confirm the applicability of the MIPs, their performance was checked in samples of spiked fruit, such as apples, bananas, oranges, and grapes, which were purchased from the local market in Nanning, Guangxi. The fruits were deep-washed, and the edible portions were homogenized. Before MIP application, the absence of ABM was confirmed from blank sample analysis. A quantity of 10 g of samples were precisely weighed and were then spiked with 10 mL of ABM methanol standard solution at concentrations of 0.05, 0.1, and 0.25 µg g^−1^. After adding 2 g of NaCl and 4 g of MgSO_4_ [43], the samples were vigorously shaken for two minutes and centrifuged for 3 min at 8000 rpm. The upper methanol layer was transferred to a vial having 40 mg of MIPs and was agitated for 30 min. The MIPs were collected with centrifugation, eluted with method 2; the extract was concentrated and reconstituted in methanol, filtered, and subjected to HPLC–UV to analyze the bound amount of ABM. 

## 3. Results and Discussion

### 3.1. Polymer Synthesis

DMSO is a suitable solvent for β-CD-based MIP production. However, neat DMSO can have a negative impact on template-monomer interactions, which could lead to lower imprinting results [44]. As RTILs are known as designer solvents, their tunable nature, in combination with DMSO, can provide a better medium for MIP synthesis [45]. 

MIPs prepared with [BMIM]BF_4_ provide more effective MIP formation when used as a co-solvent with DMSO [41]. Here, we prepared β-CD-based MIPs for ABM by using DMSO and RTILs as combined solvents in different ratios (see Table 1), and the adsorption data of the synthesized polymers is given in Figure S1. Based on these results, only the two polymers (M5 and D2) with the highest results were selected for further experiments. To confirm the effect of the RTILs, neat DMSO-based polymers were also synthesized. The polymerization ratio and polymerization time are crucial in developing MIPs [42]. Polymerization times of 4, 8, 12, 16, 20, and 24 h were first inspected, from which 8 h was found to be the ideal polymerization time to achieve desirable MIPs. The results showed that the template/monomer/cross-linker ratio of 1:6:24 provided maximum adsorption of ABM (14.55 mg g^−1^) and better selectivity when DMSO and RTIL were used in equal ratios, while, in contrast, the lowest adsorption and worst performance was observed when neat DMSO was used as a solvent. However, by increasing the RTIL ratio, the performance of the MIPs was also decreased, which could be due to the imidazolium cation (BMIM+), which interacts with the interior of the β-CD by butyl group [46]. Thus, both [BMIM]BF_4_ and β-CD at a molar ratio of 1:1 provided efficient MIPs for the selective extraction of ABA. The generalized procedure for ABM MIP synthesis is given in the graphical abstract.

### 3.2. Characterization Study

**Figure 2:**
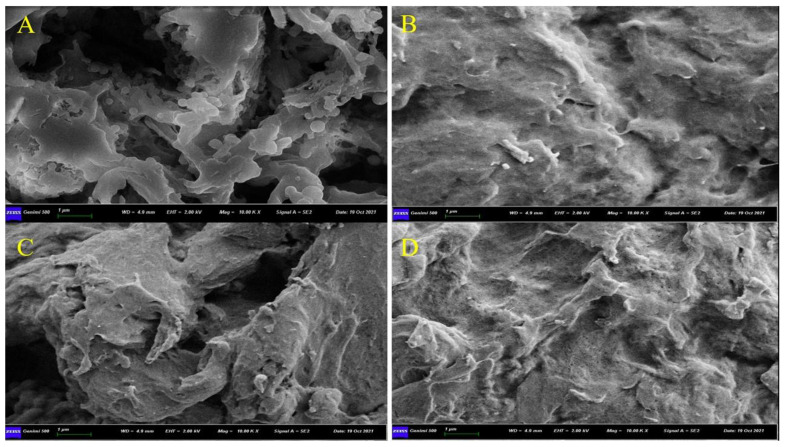
SEM images of (**A**) M5, (**B**) MN5, (**C**) D2, and (**D**) DN2.

The structural morphologies of the prepared polymers were characterized by a scanning electron microscope. As presented in Figure 2, the M5 polymers prepared by using RTILs/DMSO (1:1) provided a porous structure, as compared to the corresponding non-imprinted polymers (MN5). In comparison, the neat-DMSO-based, imprinted polymers (D2) provided a less porous structure, and obviously, its corresponding control polymer (DN2) has a compact surface, which shows that more porosity is caused by the removal of the maximum adsorbed ABM molecules [47].

**Figure 3:**
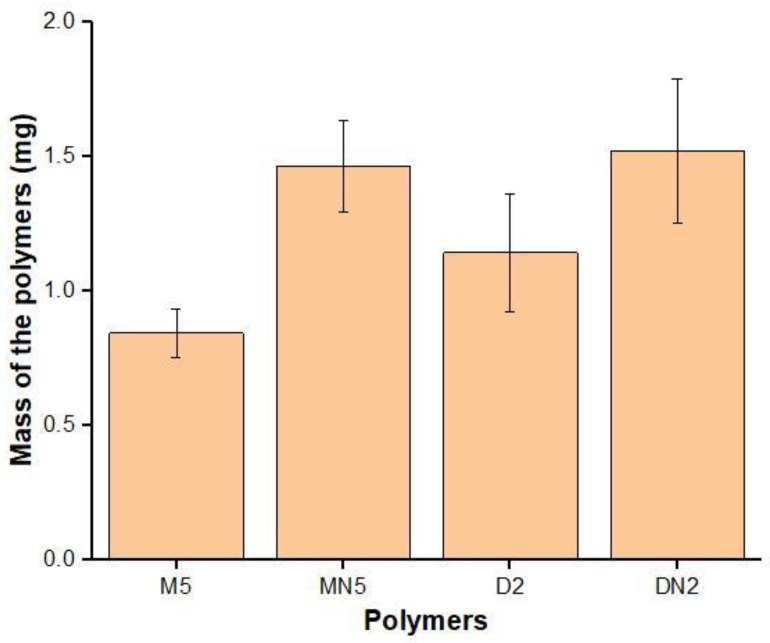
Swelling performance of the polymers.

To determine the improvement of the MIPs by using RTILs, the swelling performance of M5; D2; and their corresponding, controlled polymers was carried out in water (Figure 3). The least swelling was found in M5 when RTIL and DMSO were used as a binary solvent, followed by polymers synthesized with neat-DMSO-based MIPs, while the non-imprinted polymers showed maximum swelling. The lesser swelling indicates that the polymers had a porous structure, where the water was embedded inside the cavities, causing lesser swelling [48], indicating that M5 has a pronounced adsorption ability for ABM.

**Figure 4:**
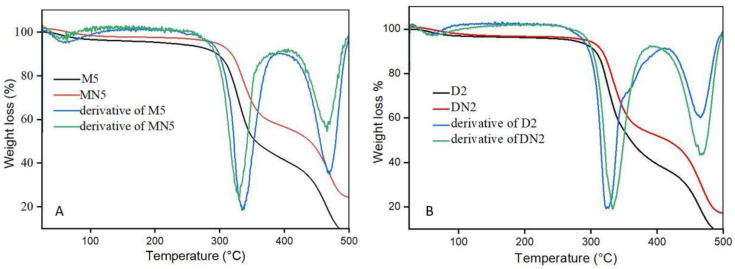
(**A**) TGA spectra of M5 and MN5, (**B**) TGA spectra of D2 and DN2.

The thermal stability of the prepared polymers was investigated with TGA, presented in Figure 4. The results revealed that weight loss was observed in three stages. First, minor weight loss was observed in the range of 50 to 100 °C, which indicates the residual water loss; the second loss occurred in the range of 300 to 350 °C, which represents the decomposition of the polymers, and the third weight loss was found after 450 °C, which contribute, to the organic matter decomposition. The TGA analysis represents that the polymers exhibited higher thermal stability up to 300 °C [49,50]. 

**Figure 5:**
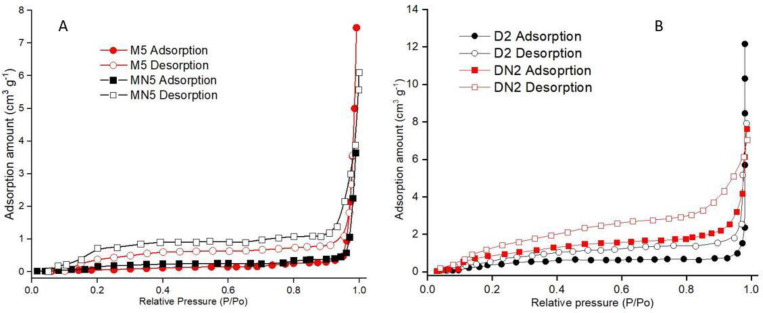
Nitrogen adsorption isotherms of (**A**) M5 and MN5, and (**B**) D2 and DN2.

A gas adsorption experiment was performed to evaluate the porosity and surface area of the prepared polymers. In Table 2, the results show that the polymers were mesoporous, as the M5 had the lowest BET surface area of 0.43 (m^2^ g^−1^) and a maximum pore volume of 23.32 (10^–3^ cm^3^ g^−1^), which was greater than both the corresponding control polymer (MN5) and also the neat-DMSO-based MIPs (D2). The highest mean pore diameter (217.43 nm) was observed in M5, while the MN5 mean pore diameter was 131.64 (nm). In contrast, the BET surface area of D2 was higher than DN2, but the D2 had an increased mean pore diameter of 81.47 (nm), while the DN2 had only 39.53 (nm) of pore diameter. Nitrogen adsorption isotherms (Figure 5) showed that the polymers were mesoporous, as they followed the “Type II” isotherms [51].

### 3.3. Binding Characteristics of the Prepared Polymers

#### 3.3.1. Adsorption Kinetics

**Figure 6:**
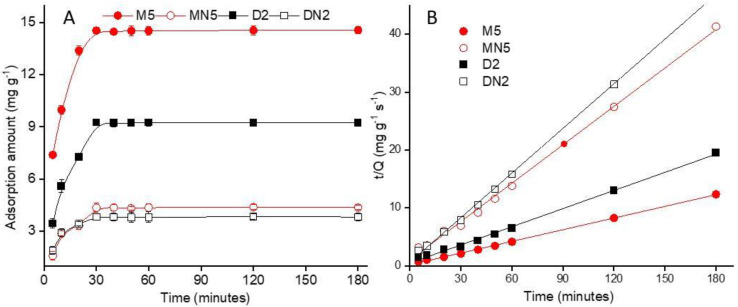
(**A**) Kinetic adsorption of the polymers; (**B**) linear fitting of the plot of *t*/*Q* vs. *t*.

The adsorption kinetics of the MIPs are given in Figure 6A. The results showed that the polymers reached a saturation point at an equilibrium binding time of 30 min. A total of 70% adsorption was first observed in 10 min with rapid adsorption in the first 5 min, which gradually reached equilibrium in a total of 30 min. To evaluate the kinetic adsorption performance, a broadly used isotherm model, the “pseudo-second-order kinetic model” was employed according to the following Equation (4):

(4) $$\frac{t}{Q_{t}}=\frac{1}{kQ_{e}^{2}}+\frac{t}{Q_{e}}\;$$

where *Q_t_* and *Q_e_* represent the ABM amount adsorbed (mg g^−1^) on polymer surface at time t, and adsorption capacity at equilibrium, respectively. The pseudo-second-order rate constant “*k*” is expressed as (mg g^−1^ s^−1^). A linear fitting plot was constructed between *t*/*Qt* versus t plot to determine the correlation coefficient (R^2^) value of the pseudo-second-order model for each polymer (Figure 6B). The results showed that M5 had the theoretical *Q_e_* value of 14.88, which was close to the experimental adsorption value. Besides the R^2^ value of M5 (0.999) was higher than MN5 and D2 (Table 3), which indicated that the adsorption process of the polymers was administered by chemisorption [52].

#### 3.3.2. Isotherm Binding

**Figure 7:**
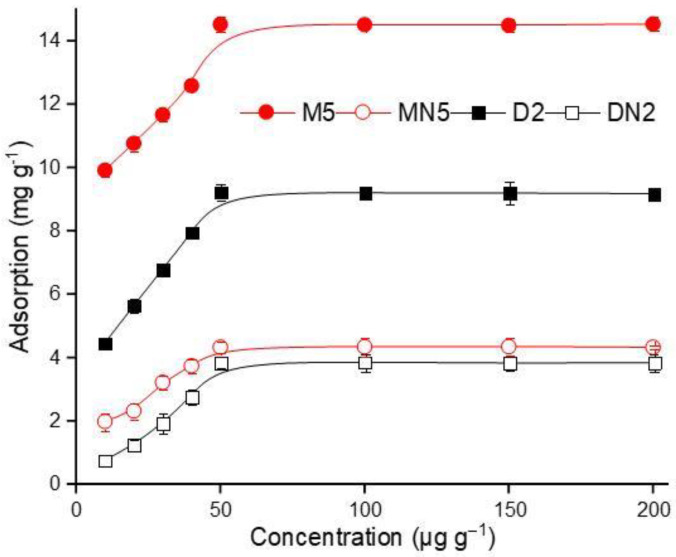
Isothermal binding of the polymers.

The isotherm binding of the polymers was evaluated by using a different concentration range of ABM standard solution, and the results are demonstrated in Figure 7. The ABM adsorption gradually increased with an increase in the concentration, which reached the equilibrium position at the final concentration of 50 µg mL^−1^. The maximum adsorption amount at the equilibrium state was 14.53 mg g^−1^ by the M5 polymers, which indicated that the polymers had a strong adoration for ABM. Langmuir (5) and Freundlich (6) isotherm models [53,54] were applied to elaborate the efficiency of the MIPs. The models are expressed as below:

(5) $$\frac{C_{e}}{Q_{e}}=\frac{1}{K_{L}Q_{max}}+\frac{C_{e}}{Q_{max}}$$

(6) $$lnQ_{e}=lnK_{f}+\frac{1}{n}lnC_{e}$$

where *C_e_* is the equilibrium concentration (µg mL^−1^) and *Q_e_* is the adsorption capacity (mg g^−1^) at equilibrium. *K_L_* and *K_f_* are Langmuir and Freundlich isotherm constants related to the affinity of the binding sites, expressed as (L mg^−1^). *Q_max_* denotes maximum adsorption capacity (mg g^−1^), which can be derived from the plot of *C_e_*/*Q_e_* versus *C_e_*. The values of the Freundlich isotherm model constants *K_f_* and *n* can be calculated from the intercept and slope of the linear plot of ln*C_e_* versus ln*Q_e_*. The results showed that the Langmuir isotherm model fitted well, as the R^2^ of M5 and D2 were 0.999 and 0.997, respectively, which were higher than both corresponding control polymers, while the R^2^ values of the Freundlich isotherm model were very low for all polymers. The *Q_max_* value of M5 is 15.08 mg g^−1^, which is 1.5 times more than D2, and 3 times more than MN5, and it has *K_L_* values of 0.03 L mL^−1^ of M5, which was also lower than all of the other polymers (Table 3). The linear fitting of both isotherm models is given in Figures S2 and S3. The presented data indicated that the polymers have monolayer chemical adsorption as the Langmuir model fitted well because of its highest R^2^ value [55].

#### 3.3.3. Selectivity Test

**Figure 8:**
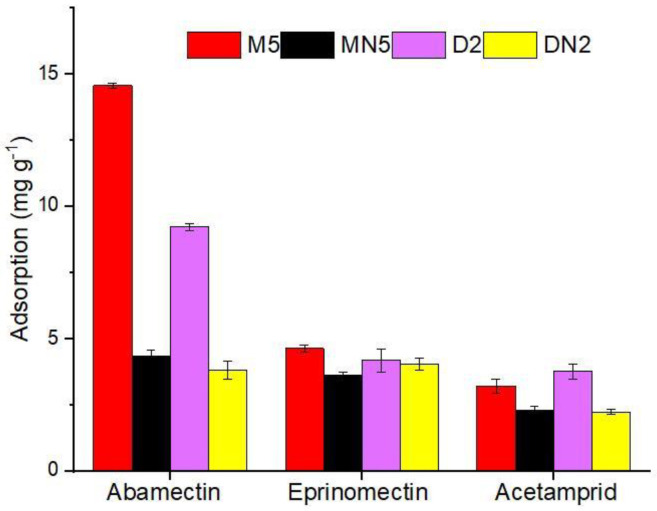
Selectivity parameters of polymers for ABM and its competitors.

To inquire about the MIPs’ performance for the selective extraction of ABM, two competitive compounds, eprinomectin and acetamiprid, were used for the recognition ability experiment. The chemical structure of the reference compounds is given in Figure 1. The selective adsorption of M5 for ABM was 3-fold higher than those of both of the competitors; similarly, the selective adsorption of D2 was 2-fold higher than those of both competitors, but both of the control polymers showed lower responses to all analytes (Figure 8). The selectivity coefficient (*SC*) (7) and imprinting factor (*IF*) (8) were used to further assist the competitive selectivity, which is expressed as shown below:

(7) $$SC=IF_{t}/IF_{a}$$

(8) $$IF=Q_{MIP}/Q_{NIP}$$

where *IF_t_* represents the template, and *IFa* refers to structural analogue imprinting factor, and the adsorption capacity of MIPs and NIPs as represented as *Q_MIP_* and *Q_NIP_* (mg g^−1^), respectively. The M5 poses *IF* of 3.36, 1.28, and 1.41 for ABM, eprinomectin, and acetamiprid, respectively, and an *SC* value of 2.62 and 2.38, respectively, in response to eprinomectin and an acetamiprid, and an almost similar trend was followed by the neat-DMSO-based polymers (Table 4). The results showed that the synthesized, imprinted polymers had high selective adsorption ability for ABM in the presence of both similar and non-similar structural analogues. 

### 3.4. Optimization Parameters

#### 3.4.1. Template Extraction

**Figure 9:**
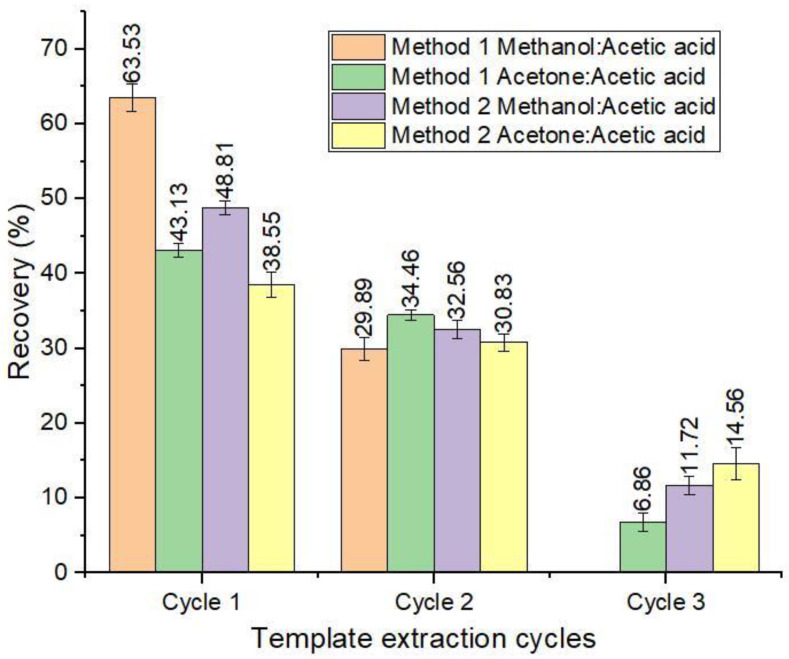
Recovery (%) of template removal with two extraction methods.

To elute the ABM molecules bound to the polymers, two types of extraction methods were investigated, and the results are presented in Figure 9. The results showed that method one (Soxhlet extraction) had the highest recovery of 93.42% in two cycles using methanol/acetic acid, while 84.45% in three cycles was achieved using acetone/acetic acid as an eluent. However, this method required more time and an increased amount of elution solvent. In contrast, method 2 had an almost equal recovery of 93.09% in three cycles using methanol/acetic acid, while about 10% less recovery was achieved in method 2 using acetone/acetic acid as the eluent. Based on these results, method 2 (ultrasonication extraction) was selected, using methanol/acetic acid as an eluent for template removal as it provides efficient elution of ABM and also exhibits lower reagent utilization and less time is required. Our results are in agreement with Kumar et al. [56], who revealed that ultrasonication extraction using methanol/acetic acid provides efficient elution of the template from the polymers. 

#### 3.4.2. Quantity of MIPs 

**Figure 10:**
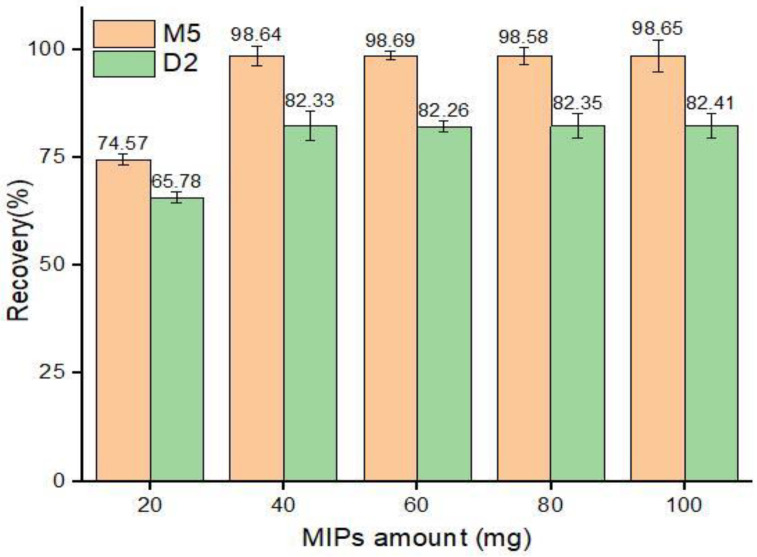
Adsorption recoveries (%) by different amounts of MIPs.

The functional monomer allows the imprinted polymers to bind the concerned analyte with hydrogen and ionic bonding, while the stability and porosity are managed by the cross-linking agents [57]. Therefore, the appropriate quantity of MIPs is important to determine to obtain efficient recovery. To investigate the optimum quantity of MIPs required, different quantities of MIPs, ranging from 20 to 100 mg, were used for sorption experiments for ABM standard solution, and their response was studied. The results (Figure 10) showed that 40 mg of both M5 and D2 polymer provided sufficient recoveries from the 5 mL of standard ABM solution of 50 µg mL^−1^, which remained constant with a further increase in sorbent amount. 

#### 3.4.3. pH Optimization

**Figure 11:**
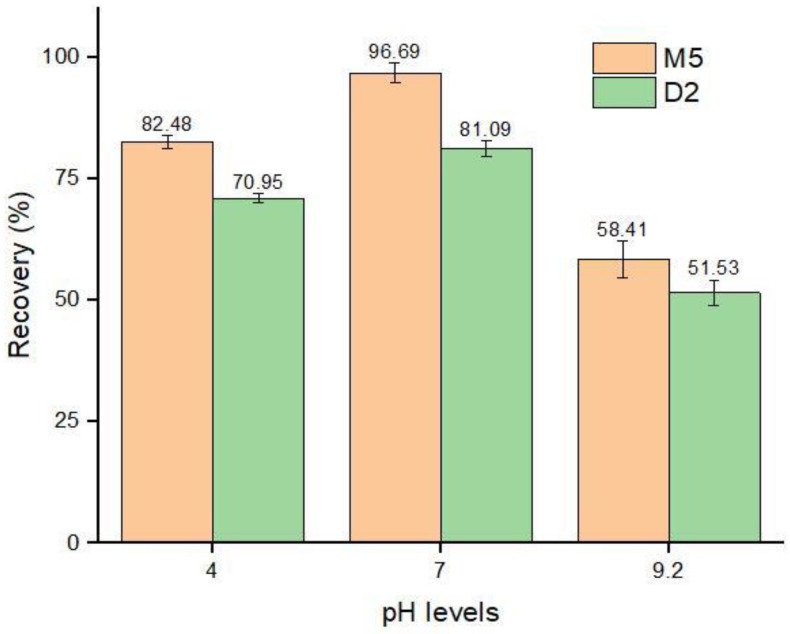
Adsorption recoveries (%) by MIPs in different pH mediums.

The extraction efficiency of the sorbents is greatly influenced by the pH of the complex sample solution [58]. The pKa value of ABM is 12.47, and it provided good recovery at a pH level of 7.0 [40]. To investigate the synthesized imprinted polymers in different pH mediums, buffer solutions of pH 4.0, 7.0, and 9.2 were fortified with an ABM standard solution of 50 µg mL^−1^ concentration. After the addition of 40 mg of MIPs, the fortified solutions were agitated for 30 min, centrifuged, and the supernatant was subjected to HPLC–UV for analysis. The results showed that the lowest recoveries, 58.41% and 51.53% by M5 and D2, respectively, were achieved at a pH level of 9.2, followed by a pH level of 4, while the highest recoveries of 96.69% and 81.09% were obtained at neutral pH levels (Figure 11). Our results are in agreement with Teixeira et al. [40], and thus the results revealed that the change in the pH medium of the sample greatly influenced the efficiency of MIPs [59].

### 3.5. Reusability of MIPs

**Figure 12:**
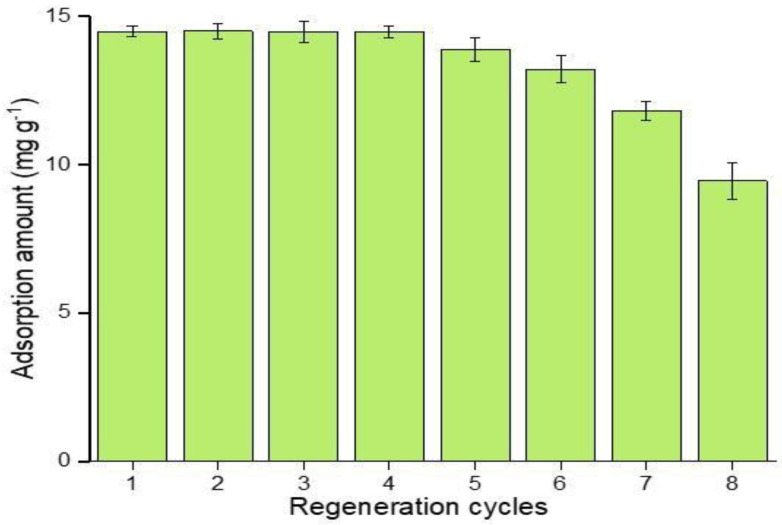
Adsorption recoveries of ABM by M5 in regenerated cycles.

Besides the selective extraction efficiency of imprinted polymers, the reusability of polymers is a key factor to elaborate its potential application, as it greatly contributes to its economical and reliable application in real-time analysis [60]. The results from Figure 12 show that, in consecutive regeneration cycles, the M5 polymer reusability was stable in the first four regeneration cycles, while in the fifth cycle, a 4.2% recovery loss was observed, which reached a 34.76% recovery loss at the eighth regeneration cycle. Thus, it is indicated that the prepared, imprinted polymers can be effectively reused, only losing 8.78% performance in six consecutive regeneration cycles. The regeneration performance of the polymers depends on the nature of inclusion interaction between host and guest molecules. 

### 3.6. Extraction of ABM from Real Samples

The application of MIPs to real complex samples is a vital step to determine its applicability for the selective extraction of ABM. For this purpose, four types of fruits with different matrices were selected, and before investigation, the method validation was performed by analyzing the standard solution of ABM, and R^2^ of 0.998 was achieved from the plot of concentration versus the calibration curve of peak area within the linear range of 0.03–1.50 µg g^−1^. The chromatogram of abamectin has been provided in the supplementary file (Figure S4). The results (Table 5) showed that the highest recovery of 101.47% in oranges, 99.20% in apples, 96.87% in grapes, and 95.33% in bananas was achieved at a spiked level of 0.25 µg g^−1^, while the lowest recovery of 81.67% was found in bananas spiked with 0.05 µg g^−1^ of ABM standard solution. The relative standard deviation (RSDs) for all of the spiked samples was in the range of 4.36–1.26%, with a limit of detection (LOD) and limit of quantitation (LOQ), calculated according to signal to noise ratio (3:10) of 0.02 µg g^−1^ and 0.05 µg g^−1^, respectively. The presented results imply that the proposed method shows good performance in real-sample application according to analytical standards.

To further elucidate the prominence of the proposed method, a comparative study was performed with the previously established methods for ABM extraction (Table 6). The proposed method presented a decent qualitative approach for the selective extraction of ABM. Based on the comparative study with the literature, the analytical parameters of the current method express its stable and selective application in real fruit samples. 

## 4. Conclusions

In this study, β-CD based MIPs were developed by using RTILs as solvents for the selective extraction of ABM from complex fruit samples. From the structural and morphological analysis, the prepared imprinted polymers showed results to valid its successful synthesis. The polymers showed a quick extraction time of 30 min with high selective adsorption for ABM in comparison to its structural analogue competitors. After optimization of certain analytical parameters, the MIPs were used to determine the ABM in real fruit samples, and the results were compared with the previously developed methods. The proposed method showed a good performance with a high recovery percentage and lower LOD and RSD values for all of the analyzed fruit samples. Additionally, the MIPs exhibit excellent selectivity, reusability, easy preparation, and good stability, thus revealing that the developed MIP-based extraction method for ABM provided good results, qualifying the analytical standards for the selective extraction of ABM, and usable as an alternative approach in comparison to traditional extraction methods for ABM.

## Figures and Tables

**Table 1:** Preparation protocol for ABM MIPs synthesis.

Polymers	ABM (Template) (mmol)	β-CD (Monomer) (mmol)	HMDI (Cross-Linker) (mmol)	RTIL (Co-Solvent) (mL)	DMSO (Solvent) (mL)	Adsorption (mg g^−1^)
D1	0.5	1.5	9	0	20	6.51
M1	0.5	1.5	9	5	15	7.79
M2	0.5	1.5	9	10	10	9.43
M3	0.5	1.5	9	15	10	8.22
D2	0.5	3	12	0	20	9.23
DN2*	0.0	3	12	0	20	3.82
M4	0.5	3	12	5	15	12.10
M5	0.5	3	12	10	10	14.55
MN5*	0.0	3	12	10	10	4.36
M6	0.5	3	12	15	10	12.81
D3	0.5	4.5	15	0	20	7.00
M7	0.5	4.5	15	5	15	7.82
M8	0.5	4.5	15	10	10	8.56
M9	0.5	4.5	15	15	10	8.13

DN2* and MN5* represent NIPs for D2 and M5, respectively.

**Table 2:** The surface area, pore volume, and pore size of the synthesized polymers.

Polymers	S_BET_ (m^2^ g^−1^)	V_p_ (10^–3^ cm^3^ g^−1^)	D_mean_ (nm)
M5	0.43	23.32	217.43
MN5	0.27	10.58	131.64
D2	11.87	14.46	81.47
DN2	3.68	9.63	39.53

**Table 3:** Pseudo-second-order, Langmuir, and Freundlich model parameters for the binding of ABM on MIPs and NIPs.

Pseudo-Second-Order Model			
Polymers	*Q_e_* (mg g^−1^)	*K* (mg g^−1^ s^−1^)	R^2^
M5	14.88	0.026	0.999
MN5	4.52	0.049	0.997
D2	9.57	0.023	0.997
DN2	3.89	0.109	0.999
**Langmuir isotherm model**			
Polymers	*Q_max_* (mg g^−1^)	*K_L_* (L mL^−1^)	R^2^
M5	15.08	0.03	0.999
MN5	4.67	0.56	0.995
D2	9.79	0.11	0.997
DN2	4.83	1.69	0.950
**Freundlich isotherm model**			
Polymers	*n*	*K*_f_ (L mg^−1^)	R^2^
M5	5.33	6.35	0.898
MN5	2.55	0.81	0.888
D2	2.86	2.06	0.913
DN2	1.26	0.13	0.913

**Table 4:** Imprinting factors and selectivity coefficients of polymers for ABM and its competitors.

Analyte	Binding Capacity (mg g^−1^)			
	M5	MN5	*IF*	*SC*
Abamectin	14.56	4.33	3.36	
Eprinomectin	4.63	3.61	1.28	2.62
Acetamiprid	3.21	2.28	1.41	2.38
	**D2**	**DN2**	***IF***	***SC***
Abamectin	9.22	3.81	2.42	
Eprinomectin	4.18	4.03	1.04	2.33
Acetamiprid	3.76	2.23	1.68	1.44

**Table 5:** Determination of ABM in fruit samples.

Sample	Spiked Levels (µg g^−1^)	Found Concentration	Recovery (%)	RSD (%)
Apple	0.05	0.047	93.87	3.44
	0.1	0.098	97.53	1.94
	0.25	0.248	99.20	1.26
Banana	0.05	0.041	81.67	3.94
	0.1	0.087	87.37	2.76
	0.25	0.238	95.33	2.50
Orange	0.05	0.044	87.07	4.17
	0.1	0.097	96.70	3.41
	0.25	0.254	101.47	2.62
Grapes	0.05	0.042	83.33	4.36
	0.1	0.091	91.37	3.48
	0.25	0.242	96.87	2.77

**Table 6:** Comparison of the present work with previously reported methods for ABM detection.

Sample	Method	Linear Range	LOD ^a^	LOQ ^b^	Recovery (%)	RSD ^c^ (%)	Reference
Edible oil	SPE-HPLC MS/MS ^d^	0.5–100 μg kg^−^^1^	0.16 μg kg^−1^	0.50 μg kg^−^^1^	91.7–101.8	2.0–7.0	[32]
Fruit juice and water	MIP ^e^-SPE-HPLC-UV	25– 750 ng mL^−1^	–	25 ng mL^−1^	88–110	1.18– 12.30	[40]
Milk and yogurt	QuEChERS ^f^ HPLC-FLD ^g^	0–25 μg L^−1^	0.1 to 3.2 μg L^−1^	0.2–10 μg L^−1^	83–112	4–10	[61]
Edible oil	LTP LC–MS/MS ^h^	5–1000 μg L^−1^	0.1–0.4 μg kg^−1^	0.3–1.3 μg kg^−1^	71.1–119.3	3.2–10.3	[62]
Apples, bananas, oranges, and grapes	MIP-HPLC-UV	0.03–1.50 µg g^−1^	0.02 µg g^−1^	0.05 µg g^−1^	81.67–101.47	1.26–4.36	This work

^a^ limit of detection; ^b^ limit of quantitation; ^c^ relative standard deviation; ^d^ solid phase extraction, high performance liquid chromatography tandem mass spectrometry; ^e^ molecularly imprinted polymers; ^f^ quick, easy, cheap, effective, rugged, and safe; ^g^ fluorescence detector; ^h^ low temperature purification, liquid chromatography–tandem mass spectrometry.

## Data Availability

Not applicable.

## Supplementary Materials

The following are available online at https://www.mdpi.com/article/10.3390/nano12061017/s1, Figure S1: Adsorption data of prepared MIPs and NIPs for ABM, Figure S2: Linear fitted plots of Ce/qe versus Ce for Langmuir, Figure S3: Linear fitted plots of log q versus log Ce for Freundlich, Figure S4: HPLC-UV chromatograph for abamectin, eprinomectin and acetamiprid, Table S1: Details of polymerization components.
