# Supplementary material for: Room-Temperature, Ionic-Liquid-Enhanced, Beta-Cyclodextrin-Based, Molecularly Imprinted Polymers for the Selective Extraction of Abamectin
Source: Nanomaterials (Basel). 2022 Mar 20;12(6):1017. doi: 10.3390/nano12061017 (PMC8953458; doi:10.3390/nano12061017)
Supplement: Supplementary file 1 [file nanomaterials-12-01017-s001.zip › nanomaterials-1603006-supplementary.pdf]

# Room-Temperature, Ionic-Liquid-Enhanced, Beta-Cyclodextrin-Based, Molecularly Imprinted Polymers for the Selective Extraction of Abamectin

**Saqib Farooq [1], Bochang Chen [1], Shakeel Ahmad [1], Ihsan Muhammad [1], Quaid Hussain [2] and Haiyan Wu [1,*]**

1 Guangxi Key Laboratory of Agric-Environment and Agric-Products Safety, Agricultural College of Guangxi University, Nanning 530004, China; saqibhort@gmail.com (S.F.); 15677802394@163.com (B.C.); shakeel1287@hotmail.com (S.A.); ihsanagrarian@yahoo.com (I.M.)
2 State Key Laboratory of Subtropical Silviculture, Zhejiang A&F University, 666 Wusu Street, Hangzhou 311300, China; quaid_hussain@yahoo.com
* Correspondence: whyzxb@gmail.com

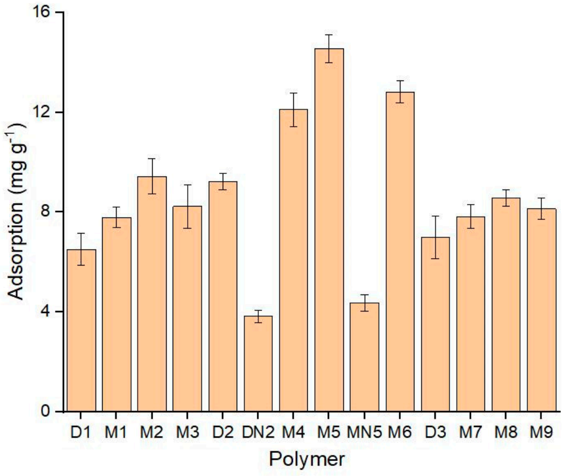


**Figure S1.** Adsorption data of prepared MIPs and NIPs for ABM

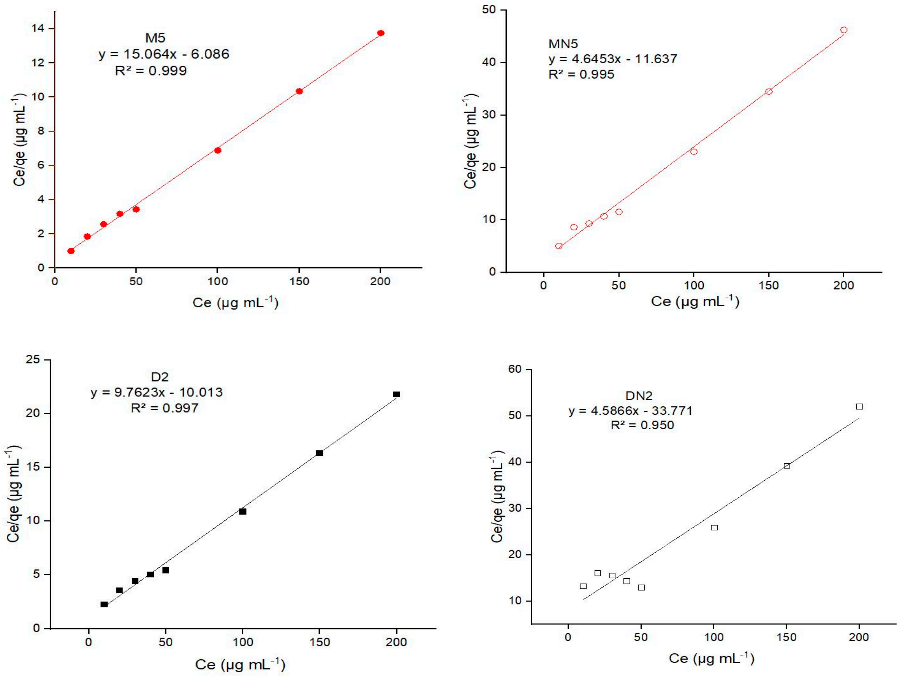


**Figure S2.** Linear fitted plots of *Ce*/*q*e versus Ce for Langmuir

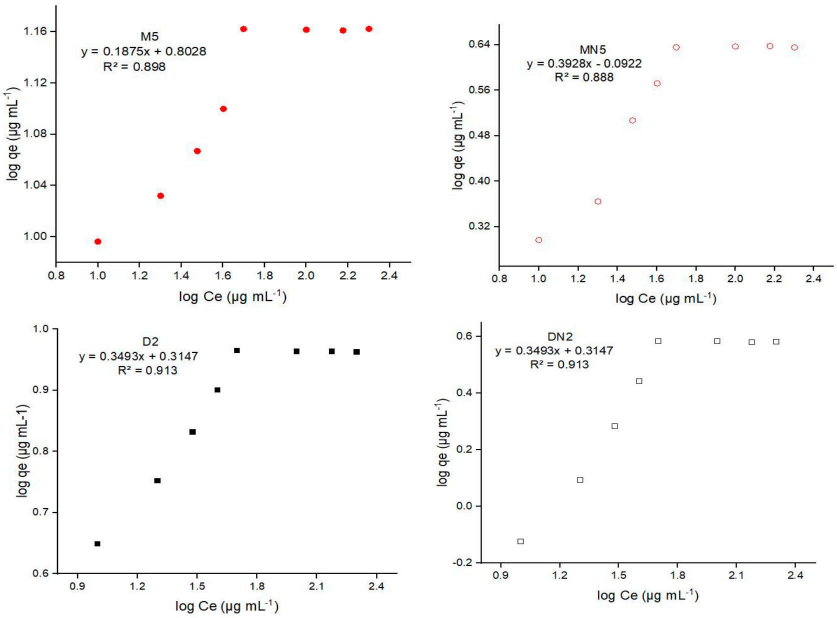


**Figure S3.** Linear fitted plots of log q versus log Ce for Freundlich

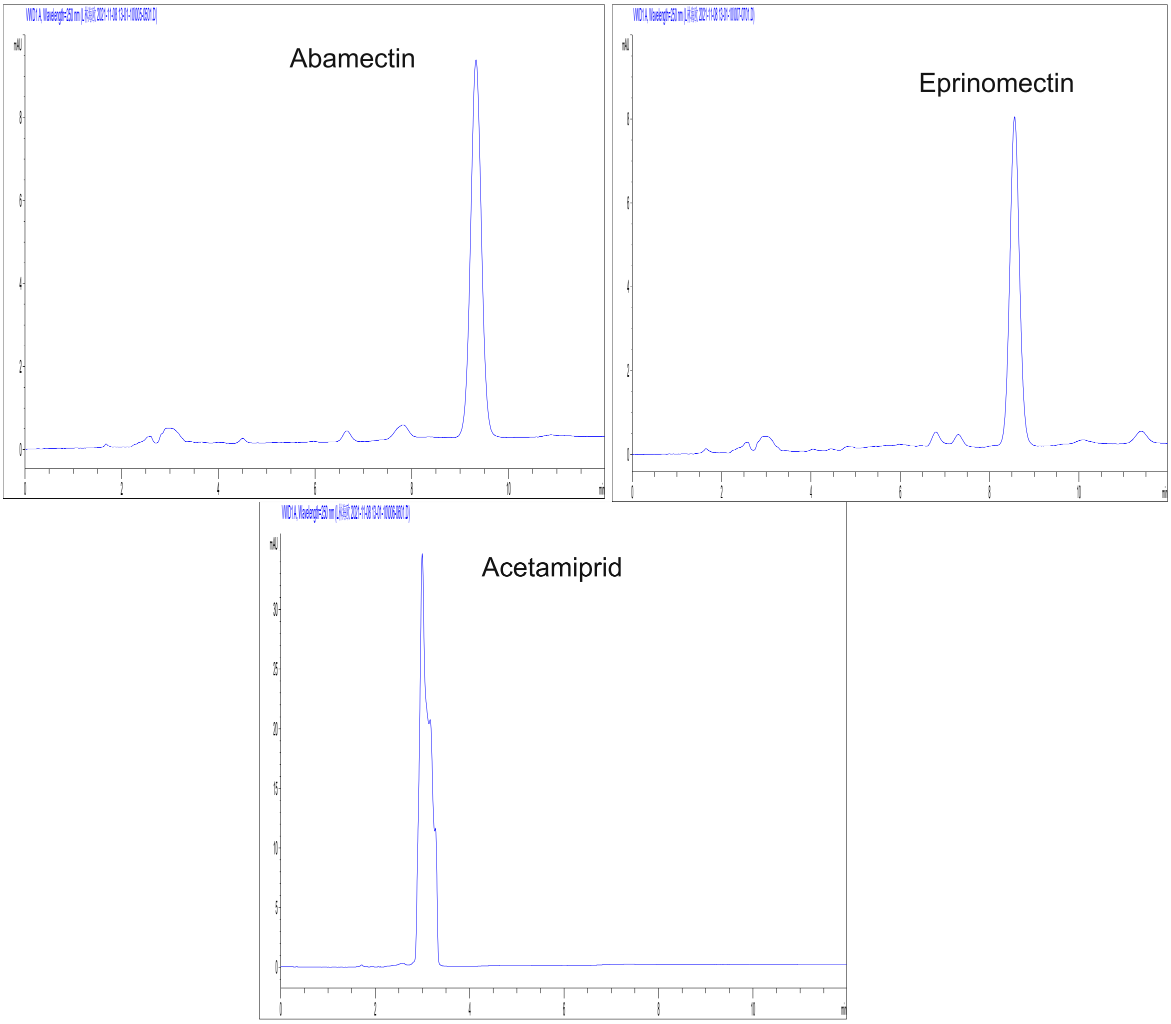


**Figure S4.** HPLC-UV chromatograph for abamectin, eprinomectin and acetamiprid.

Table S1: Details of polymerization components

| Name | Molecular Formula | Molecular weight | Role |
|---|---|---|---|
| Abamectin | $C_{49}H_{74}O_{14}$ | 887.11 | Template |
| 1-butyl-3-methylimidazolium tetrafluoroborate | $C_8H_{15}BF_4N_2$ | 226.02 | Solvent |
| Dimethyl sulfoxide | $C_2H_6OS$ | 78.13 | Solvent |
| 1,6-hexamethylene diisocyanate | $C_8H_{12}N_2O_2$ | 168.19 | Cross-linker |
| Beta-cyclodextrin | $C_{42}H_{70}O_{35}$ | 1134.99 | Functional monomer |
